# Genome-Wide Pleiotropy Analysis Identifies Shared and Opposing Pathways Influencing Coronary Artery Disease and Cancer

**DOI:** 10.1161/ATVBAHA.125.322433

**Published:** 2025-08-28

**Authors:** James Yarmolinsky, Evelyn Lau, Fotios Koskeridis, Marc J. Gunter, Dennis Wang, Abbas Dehghan, Ioanna Tzoulaki

**Affiliations:** Department of Epidemiology and Biostatistics, School of Public Health (J.Y., F.K., M.J.G., A.D., I.T.), Imperial College London, United Kingdom.; National Heart and Lung Institute (E.L., D.W.), Imperial College London, United Kingdom.; Dementia Research Institute (A.D., I.T.), Imperial College London, United Kingdom.; Institute for Human Development and Potential, Agency for Science, Technology and Research (A*STAR), Singapore, Republic of Singapore (D.W.).; Bioinformatics Institute, Agency for Science, Technology and Research (A*STAR), Singapore, Republic of Singapore (D.W.).; Biomedical Research Foundation Academy of Athens, Greece (I.T.).

**Keywords:** blood pressure, cardiovascular diseases, comorbidity, coronary artery disease, risk factors

## Abstract

**BACKGROUND:**

Coronary artery disease (CAD) and cancer are 2 leading global causes of mortality, with shared modifiable risk factors, yet the genetic and molecular mechanisms underlying their comorbidity remain poorly understood.

**METHODS:**

We performed a genome-wide pleiotropy analysis to identify shared genetic mechanisms across CAD and 4 common cancers that share modifiable risk factors with CAD (breast, colorectal, lung, prostate).

**RESULTS:**

Using genome-wide pleiotropy and colocalization analysis, we identified 60 colocalized susceptibility loci shared by CAD and site-specific cancer, of which 43 are novel, including loci at *TERT*, *MYO9B*, and *SREBF1*. For 35 loci, the lead SNP (single-nucleotide polymorphism) exhibited opposing effects on CAD and cancer risk. Gene-set enrichment analysis revealed distinct enrichment patterns of same-direction and opposing-direction pleiotropic loci, including differential associations with blood pressure–related traits, blood cell traits, and waist circumference. By integrating transcriptomic and proteomic data in multitrait colocalization, 13 pleiotropic loci influenced CAD and cancer risk via differential gene or protein expression of neighboring genes, including *CALCRL*, *ANGPTL4*, and *LAMC1*, targets of approved or investigational medications. Phenome-wide association analysis in the UK Biobank identified 1955 associations (false discovery rate [FDR] *P*<0.05) of lead SNPs at multitrait colocalized loci with serum biomarkers and clinical measures, with apoA, HDL (high-density lipoprotein) cholesterol, and creatinine being associated with the largest number of lead SNPs.

**CONCLUSIONS:**

Our findings highlight shared and opposing genetic loci between CAD and cancer and provide insight into molecular intermediates mediating joint disease risk. Importantly, they indicate potential drug repurposing opportunities for dual CAD and cancer prevention while highlighting possible adverse and divergent effects of existing medications across both conditions.

**Highlights**

- Our genome-wide pleiotropy and genetic colocalization analyses identified 60 joint susceptibility loci influencing coronary artery disease and cancer (breast, colorectal, lung, or prostate), with the majority of loci conferring effects in opposing directions across diseases.
- Gene set enrichment and tissue-specificity analyses identified distinct biological pathways enriched among joint susceptibility loci with same-direction and opposing-direction effects, including differential effects on blood pressure, blood cell traits, and waist circumference.
- Multitrait colocalization integrating proteogenomic and transcriptomic data identified 13 joint susceptibility loci that influenced coronary artery disease and cancer risk via differential gene or protein expression, including *CALCRL*, *ANGPTL4*, and *LAMC1*, targets of approved or investigational medications.
- Phenome-wide association analysis in the UK Biobank associated these multitrait colocalized pleiotropic loci with a large number of clinical traits and serum biomarkers, including apoA, HDL (high-density lipoprotein) cholesterol, and creatinine, providing further insight into mechanisms contributing to coronary artery disease and cancer comorbidities.

Cancer and cardiovascular disease (CVD) are the 2 leading causes of death worldwide.^1^ Although historically considered as discrete disease entities with distinct causes, emerging evidence suggests a multifaceted and bidirectional link between these conditions.^2–6^ For example, individuals with CVD have an increased risk of subsequently developing cancer, and adult cancer survivors are at elevated risk of CVD.^2,3,7,8^

Several hypotheses have been proposed to explain these observations.^9–11^ Treatment for one disease may lead to an increased risk for the other. For example, cancer treatments can induce cardiotoxicity, leading to cardiomyopathy and heart failure.^12^ Additionally, shared modifiable risk factors across CVD and cancer (eg, tobacco use, obesity, type 2 diabetes) suggest overlapping pathophysiological mechanisms contributing to both conditions.^9,10^ This is supported by evidence that chronic inflammation, metabolic dysregulation, and clonal hematopoiesis of indeterminate potential can increase the risk of CVD and cancer.^10,13^ In addition, clinical trials have demonstrated that therapeutic inhibition of key inflammatory mediators, such as COX-2 and interleukin-1β, can jointly lower the risk of CVD and site-specific cancer.^14–17^ Despite some efforts to unravel the mechanistic links between these conditions, the key genetic and molecular pathways they share remain poorly understood.^18^ Enhanced understanding of these shared mechanisms could facilitate development of integrated preventive strategies and improved pharmacological management of CVD-cancer comorbidities.^19^

Here, we aimed to comprehensively study the genetic liability to coronary artery disease (CAD)—the most prevalent form of CVD—and 4 common adult cancers (breast, colorectal, lung, prostate) that share modifiable risk factors with CAD. Our aim was to identify shared genetic and molecular mechanisms underlying these conditions. To achieve this, we performed a genome-wide pleiotropy analysis and subsequent genetic colocalization to identify shared genetic variants. To further characterize the biological significance of these findings, we then applied a comprehensive set of in silico approaches, including multitrait colocalization integrating proteomic and transcriptomic data, phenome-wide association study (PheWAS) analysis, *cis*-Mendelian randomization, gene-set enrichment, tissue-specificity analysis, and druggability and drug repurposing analysis.

## Methods

The authors declare that all supporting data are available within the article and the Supplemental Material. The data sets used in this study are listed in Major Resources Table in the Supplemental Material. This article follows the Strengthening the Reporting of Genetic Association Studies reporting guidelines.^20^

### Study Populations

Summary genetic association data on 181 522 CAD cases among 1 165 690 participants were obtained from a genome-wide association study (GWAS) of the CARDIoGRAMplusC4D Consortium.^21^ Summary genetic association data were also obtained from GWAS of 4 cancer outcomes (breast, colorectal, lung, prostate) in up to 320 271 cases and 338 488 controls.^22–25^ A summary of case and control numbers across each cancer outcome, along with sex and demographic information for CAD and each site-specific cancer GWAS is presented in Table S1. All analyses were restricted to individuals of or predominantly of European ancestry and were further adjusted for principal components of genetic ancestry. Further information on statistical analysis, imputation, and quality control measures for summary genetic association data obtained from cancer consortia is available in the original publications. All studies contributing data to these analyses had the relevant institutional review board approval from each country, in accordance with the Declaration of Helsinki, and all participants provided informed consent.

Tissue-specific gene expression analyses were performed using summary genetic association data on *cis*-expression quantitative trait loci (eQTL) from tissue samples in up to 838 donors of predominantly European ancestry (85.3%) in Genotype-Tissue Expression V8 project.^26^ Whole-blood gene expression analyses were performed using summary genetic association data on *cis*-eQTL from 31 684 individuals of predominantly European ancestry in eQTLGen Phase I.^27^ Plasma protein expression analyses were performed using summary genetic association data on *cis*-protein quantitative trait loci (pQTL) from 35 559 individuals of Icelandic ancestry in the deCODE study.^28^ We used pQTL data from the deCODE study, as opposed to using pQTL data from a previous GWAS of plasma proteins in the UK Biobank by Sun et al^29^ to (1) minimize issues of sample overlap of UK Biobank participants with cancer and CAD susceptibility GWAS that could have arisen had we used pQTL data from Sun et al^29^ and (2) to maximize the number of proteins that could be included in multitrait colocalization analyses given the greater protein coverage on the SomaScan version 4 assay (N=4907 proteins) used in the deCODE study as compared to the Olink Explore 3072 proximity extension assay (N=2923) used in Sun et al.^29^

### Statistical Analysis

#### Genome-Wide Pleiotropy Analysis

We used pleiotropic analysis under a composite null hypothesis (PLACO) to identify pleiotropic loci that jointly influence liability to CAD and site-specific cancer in pairwise analyses.^30^ In brief, PLACO tests the composite null hypothesis that a variant is associated with none or only 1 of 2 traits. The test statistic is generated as the product of the *Z* statistic of the association of each variant with each trait (*Z*^2^), which is assumed to follow a mixture distribution. Before analysis, we removed all variants with a minor allele frequency <0.01 and a Z^2^>80 to minimize bias due to extremely large effect sizes. To account for modest sample overlap across studies, we calculated the correlation between Z scores for the subset of SNPs (single-nucleotide polymorphisms) with no effect on either trait (defined as *P*≥10^−4^ for both traits) for all CAD-cancer pairwise analyses. We then decorrelated the Z scores by multiplying them by the inverse square root of their correlation matrix.

### Functional Mapping and Annotation

For all variants with strong evidence of having pleiotropic effects on CAD and site-specific cancer (*P*_PLACO_<5×10^−8^), we then identified distinct pleiotropic loci by clumping variants in a ±500 kb radius and linkage disequilibrium (LD) thresholds set at *r*^2^=0.6 and *r*_2_^2^=0.1 using the 1000 genomes phase 3 reference panel into a single genetic locus using FUMA (SNP2GENE function, v1.3.6a), as suggested by the developers of PLACO.^31^ Lead SNPs at clumped loci were then mapped to protein-coding genes in FUMA based on positional (10 kb maximum distance to map genes) information.

### Colocalization

Colocalization was performed to provide evidence of shared causal variants at pleiotropic loci associated with CAD and site-specific cancer, thus ensuring that pleiotropic loci were not driven by lead SNPs having extremely large effects on one trait and small or null effects on the other trait.^32^ We used the coloc package to generate posterior probabilities that associations between liability to CAD and site-specific cancer represent each of the following configurations: (1) neither CAD nor site-specific cancer has a genetic association in the region (H_0_), (2) only CAD has a genetic association in the region (H_1_), (3) only cancer has a genetic association in the region (H_2_), (4) both CAD and cancer have a genetic association in the region but have different causal variants (H_3_), and (5) both CAD and cancer have a shared causal variant in the region (H_4_). Colocalization was performed by generating ±200 kb windows around the lead SNP in each pleiotropic locus. We employed default priors for p1 (ie, prior probability that a variant is associated with CAD, 1×10^−4^), p2 (ie, prior probability that a variant is associated with cancer, 1×10^−4^), and p12 (ie, prior probability that a variant is associated with both traits, 1×10^−^^5^). We used a posterior probability of colocalization (PPH_4_)>50% to indicate support for colocalization of CAD and cancer associations at each locus, as this represents the posterior probability with the majority support across all configurations tested. Among pleiotropic loci with majority support for colocalization, reported posterior probabilities represent a continuum of support for shared causal variants and can, therefore, be interpreted as providing different degrees of evidence in favor of colocalization.

### Identification of Novel Joint CAD and Cancer Susceptibility Loci

For all genes mapped to colocalized pleiotropic loci, we explored if loci in or in proximity to these genes have previously been mapped to both CAD and site-specific cancer using LDlinkR. We defined loci as novel joint susceptibility loci if there was no SNP within ±500 kb or LD *r*^2^>0.20 from the lead SNP in the locus previously reported to be associated with both CAD and relevant site-specific cancer in the GWAS Catalog (as of October 15, 2024).

### Protein-Protein Interaction Analysis

We then performed protein-protein interaction network analysis using the STRING database v11 to explore the interaction of genes mapping to colocalized pleiotropic loci across each cancer site.^33^ STRING systematically collects and integrates protein-protein interactions, including both physical interactions as well as functional associations. Searches were performed using the full STRING network, a medium confidence score (0.400), and a 5% FDR threshold.

### Gene-Set Enrichment Analysis

We also performed various gene-set enrichment analyses for all genes mapped to colocalized pleiotropic loci using the GENE2FUNC function in FUMA. We restricted analyses to protein-coding genes using Ensembl version 102 and used gene expression data on 30 tissues included in Genotype-Tissue Expression v8. We compared the enrichment of gene sets for genes mapped to lead SNPs at colocalized pleiotropic loci to genes mapped to loci strongly associated with CAD (*P*<5×10^−8^) to identify biological pathways that are uniquely shared across liability to CAD and cancer using pathways described in Hall Mark, KEGG (Kyoto Encyclopedia of Genes and Genomes), Reactome, and GWAS catalog gene sets. Selected pathways were those with a minimum of 2 overlapping genes with gene sets and those enriched at an FDR *P*<0.05 threshold.^34^ We also explored tissue-specific expression patterns of genes mapped to both CAD and cancer and compared these patterns to genes exclusively mapped to CAD in the CARDIoGRAMplusC4D Consortium GWAS to identify potential differences in tissue expression patterns across both groups. We employed the same (previously described) parameters in FUMA for gene mapping and gene-set enrichment analysis for pleiotropic loci influencing CAD and cancer to loci exclusively associated with CAD.

### Multitrait Colocalization and *Cis*-Mendelian Randomization Analysis

For colocalized pleiotropic loci, we explored if shared genetic signals across CAD and cancer were mediated through differential expression of neighboring genes or their protein products using HyprColoc multitrait colocalization.^35^ We restricted analyses to genes mapped to either lead variants or SNPs in high LD (*r*^2^>0.80) with lead variants at colocalized pleiotropic loci that also had tissue-specific or whole-blood expression *cis*-eQTL or plasma *cis*-pQTL associated (*P*<5×10^−8^) with expression of the relevant gene or protein, respectively. In Genotype-Tissue Expression V8, we restricted analyses to CAD and site-specific cancer-relevant tissues (adrenal gland, aorta artery, coronary artery, liver, lung, mammary breast, prostate, sigmoid colon, subcutaneous adipose, tibial artery, transverse colon, and visceral omentum adipose). Multitrait colocalization was performed by generating ±200 kb windows around the transcription start site of each gene evaluated. HyPrColoc was performed using default variant-specific prior configuration; priors 1 and 2 were set at 1×10^−4^ and 0.02, respectively; and regional and alignment thresholds of 0.5 were used. We used a posterior probability of colocalization >50% to indicate support for colocalization of CAD, cancer, and putative molecular intermediates at each locus.

For pleiotropic loci with evidence of multitrait colocalization (ie, colocalization across CAD, cancer, and a molecular intermediate), we estimated the effect of the molecular intermediate (ie, gene or protein expression) on risk of CAD and site-specific cancer using *cis*-Mendelian randomization.^36^ We constructed genetic instruments for each molecular intermediate using the lead SNP at each colocalized pleiotropic locus. We used the Wald ratio to generate effect estimates and the delta method to approximate SEs.^37^ We tested the relevance Mendelian randomization assumption by calculating F statistics for all genetic instruments.^38^ We minimized the likelihood of violations of the exchangeability assumption by using summary genetic association data restricted to individuals of European ancestry with further adjustment for principal components of genetic ancestry.^39^ Violations of the exclusion restriction assumption were minimized by employing colocalization analysis to rule out confounding by LD.^40^

### Phenome-Wide Association Study Analysis

To explore mechanisms underpinning the effects of multitrait colocalized pleiotropic loci, we performed a PheWAS of the lead SNP at each locus using data on 48 serum biomarkers, anthropometric traits, and clinical measures in the UK Biobank. These included all 30 blood biochemistry measures, 3 anthropometric traits (ie, body mass index, hip circumference, waist circumference), and 15 clinical measures (systolic and diastolic blood pressure, 13 blood cell traits) hypothesized to influence CAD and cancer that were measured at baseline. In post hoc analyses, we also included measures of 2962 plasma proteins that met quality control assessment. The UK Biobank is a prospective cohort study of 502 507 participants from England, Scotland, and Wales who were recruited between 2006 and 2010. Details of genotyping quality control, phasing, and imputation in the UK Biobank are described elsewhere.^41^ All traits were inverse-normal rank-transformed before analysis. Multivariable linear regression models were adjusted for age, sex, genotyping array, assessment center, and the first 10 principal components of genetic ancestry. PheWAS were performed using PHESANT.^42^ We employed a Benjamini-Hochberg FDR *P*<0.05 correction to account for multiple testing across each analysis.

### Evaluation of Drug Repurposing and Druggability of Proteins Encoded by Genes Mapped To Multitrait Colocalized Loci

Finally, for genes mapped to colocalized pleiotropic loci, we explored their druggability using data from Finan et al.^43^ This analysis combined data on protein targets of known and investigational medications, proteins with sequence similarities to these targets, and secreted or extracellular proteins belonging to druggable families, to identify 4479 genes that are drugged or considered druggable. We also searched the Open Targets platform, the Therapeutic Target Database, and ClinicalTrials.gov to identify approved or investigational medications targeting these genes. The availability of drugs targeting these genes could suggest the potential for their repurposing as pharmacological agents for joint CAD and cancer prevention.

**Figure 1.**
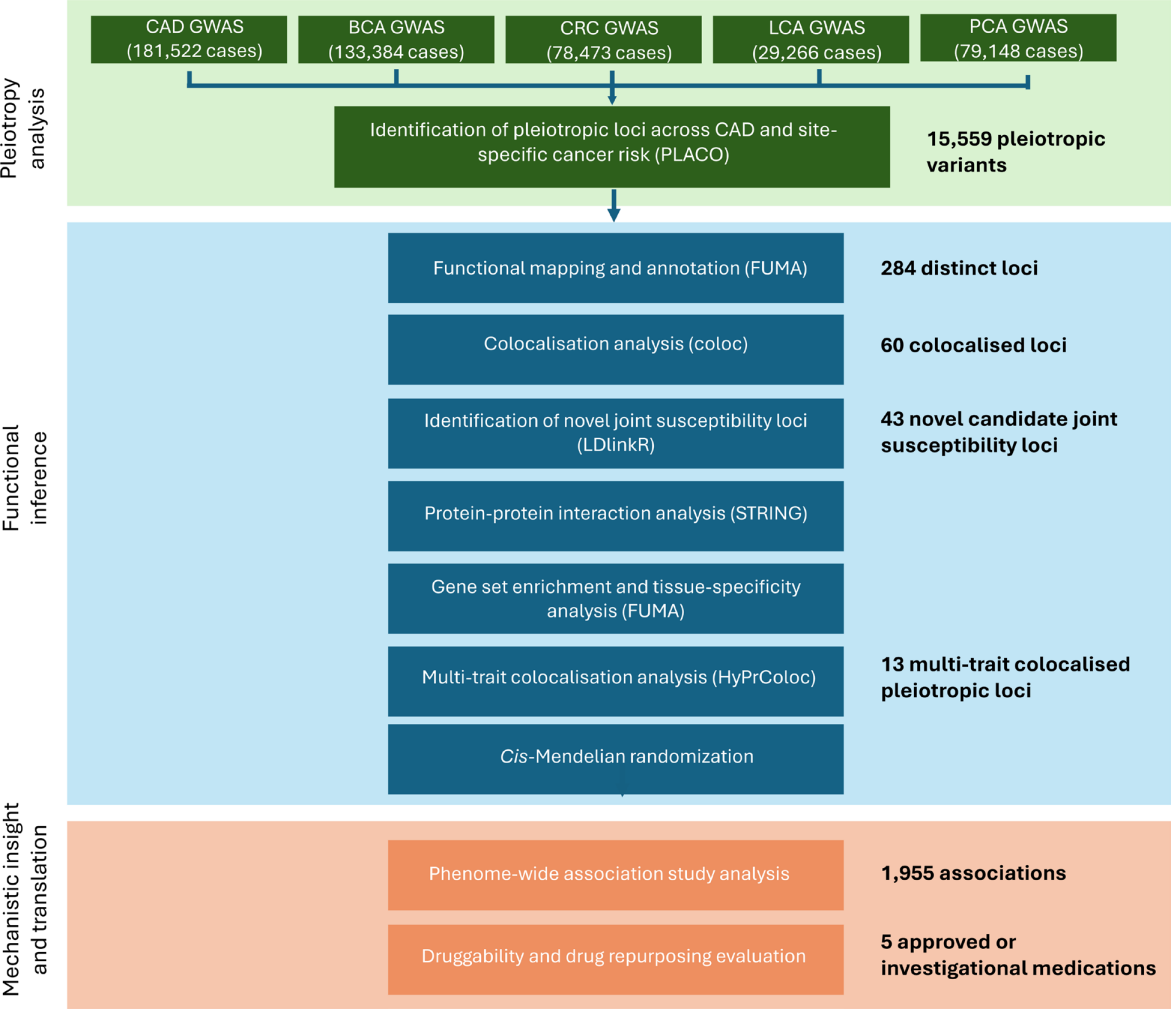
**Schematic overview of the analytical stages of this study.** BCA indicates breast cancer; CRC, colorectal cancer; FUMA, Functional Mapping and Annotation of Genome-Wide Association Studies; GWAS, genome-wide association study; LCA, lung cancer; PCA, prostate cancer; and PLACO, pleiotropic analysis under a composite null hypothesis.

A schematic overview of the analysis plan for this study is presented in Figure 1.

## Results

### Identification of Pleiotropic Loci Associated With CAD and Cancer

**Figure 2.**
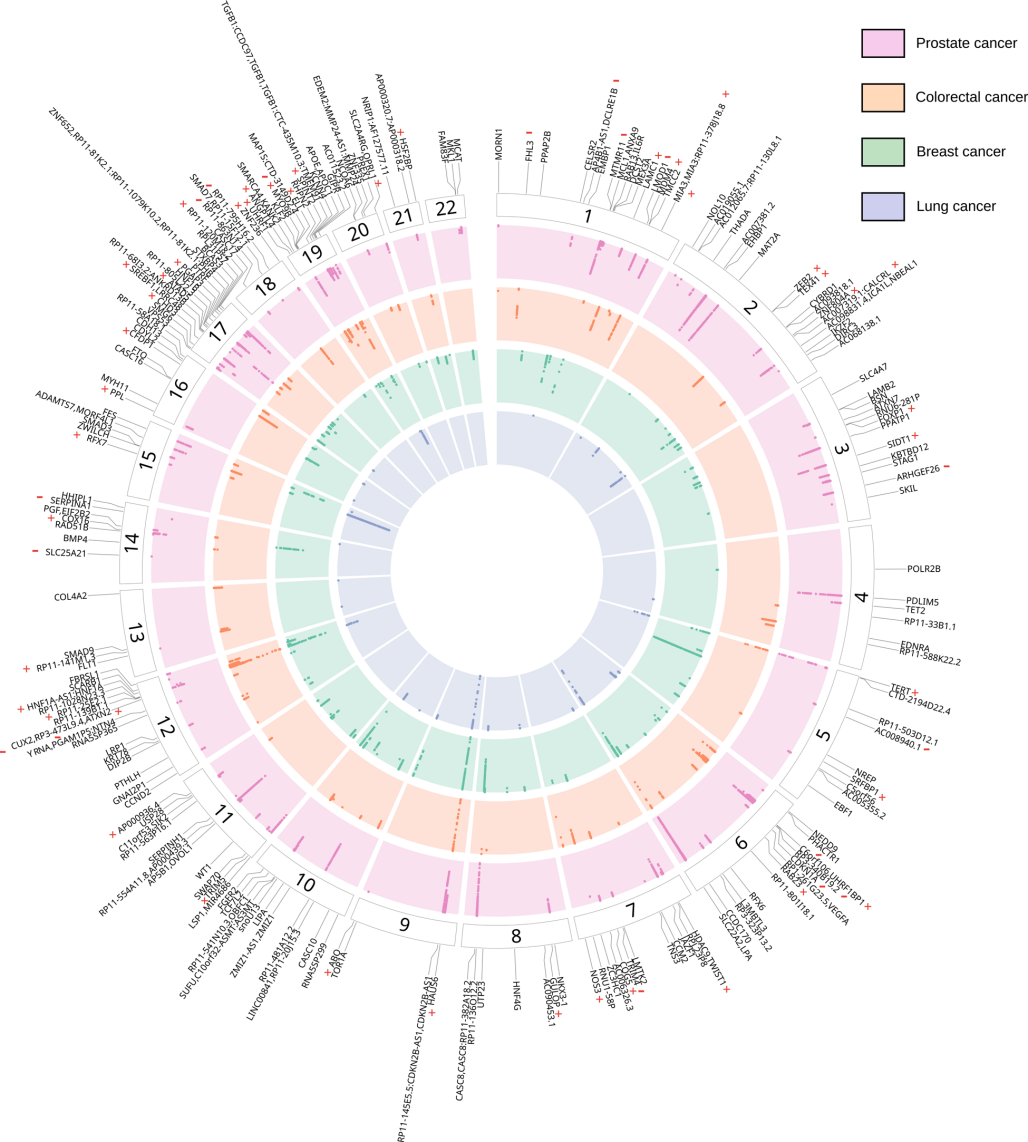
**Circular plot with pleiotropic analysis under composite null hypothesis results for analyses examining pleiotropic loci for coronary artery disease and breast cancer, colorectal cancer, lung cancer, and prostate cancer.** + and − represent pleiotropic loci with colocalization evidence where lead SNPs have effects on coronary artery disease (CAD) and cancer risk in the same direction or opposite direction, respectively.

In PLACO, 15 559 pleiotropic SNPs were identified across CAD and at least 1 of the 4 cancer sites (2798 for breast cancer, 4217 for colorectal cancer, 1069 for lung cancer, and 7474 for prostate cancer). After clumping these SNPs in FUMA, there were 284 distinct loci (86 for breast cancer, 69 for colorectal cancer, 27 for lung cancer, 102 for prostate cancer; Figure 2; Tables S2 through S5).

Colocalization analysis of the genomic region centered on the lead SNP of those 284 distinct pleiotropic loci provided evidence for a shared causal variant for liability to CAD and cancer (PPH_4_ > 50%) at 60 colocalized loci (17 for breast cancer, 20 for colorectal cancer, 2 for lung cancer, 21 for prostate cancer). One locus was shared across liability to CAD, colorectal, and prostate cancer (lead SNP in locus: rs7246865). Lead SNPs at colocalized pleiotropic loci mapped to 60 genes and variants in high LD (*r*^2^>0.80), with these SNPs mapped to an additional 73 genes. For 35 loci, the lead SNP in the locus was associated with liability to CAD and cancer with opposing directions of effect (Table S6).

Only 17 of the 60 colocalized pleiotropic loci have previously been linked to both CAD and the relevant site-specific cancer and, consequently, considered joint disease susceptibility loci in the GWAS Catalog. These loci mapped to genes including *CDKN1A* (breast, CAD), *FHL3* (colorectal, CAD), and *SERPINA1* (prostate, CAD). Consequently, 43 of 60 colocalized pleiotropic loci were considered novel candidate joint susceptibility loci (Table S6).

### Integrative Genomic and Functional Analyses Highlight Distinct Tissue-Specific and Trait-Associated Signatures of Joint CAD and Cancer Susceptibility Genes

Among 133 genes mapped to lead SNPs at 60 colocalized pleiotropic loci or variants in high LD (*r*^2^>0.80) with these SNPs, 6 had evidence of being interacting genes for breast cancer (*APOC3*-*COG5*, *CDKN1A*-*TERT*, *DCLRE1B*-*TERT*, and *ZEB2*-*TERT*), 6 for colorectal cancer (*ANGPTL4*-*LOX*, *ERBB2*-*LOX*, *NOS3*-*LOX*, and *SF3A3*-*SNRPC*), and 10 for prostate cancer (*ATPAF2*-*DRC3*, *CHD3*-*RAI1*, *HDAC9*-*MIA3*, *MYO9B*-*SH2B3*, and *PPL*-*SPINT2*; Figures S1 through S3). For example, p21, encoded by *CDKN1A*, has been shown to mediate the effect of telomere shortening, inhibited by telomerase reverse transcriptase and encoded by *TERT*, on cellular senescence.^44^ Complete results from STRING analyses are presented in Tables S7 through S9.

In gene-set enrichment analysis, the 60 genes mapped to lead SNPs at 60 colocalized pleiotropic loci were strongly enriched in GWAS catalog gene sets for various traits including lipids (eg, HDL cholesterol, apo A_1_), visceral adiposity measures (eg, waist circumference adjusted for body mass index), and blood pressure measures (eg, systolic blood pressure, diastolic blood pressure, pulse pressure). When compared with CAD susceptibility genes identified in a recent CAD GWAS of 181 522 cases and 984 168 controls that were not also mapped to lead SNPs at colocalized pleiotropic loci,^21^ candidate joint CAD/cancer susceptibility genes were more likely to be enriched for genes implicated in red blood cell traits (ie, mean corpuscular volume, mean platelet volume). Complete gene-set enrichment analysis findings for candidate joint susceptibility genes for CAD and site-combined and site-specific cancer are presented in Tables S10 through S13 (gene-set enrichment analysis was not performed for CAD and lung cancer because only 1 gene was mapped to colocalized pleiotropic loci). Findings from analyses of other annotated gene sets in the Molecular Signatures Database, curated gene sets, and WikiPathways are presented in Table S14.

When exploring tissue-specificity of gene expression, we also found distinct patterns of tissue expression for candidate susceptibility genes jointly mapped to CAD and cancer as compared to those exclusively associated with CAD (Tables S15 and S16).

### Distinct Enrichment Patterns and Tissue-Specific Expression of Genes Mapped to Same-Direction and Opposing-Direction Lead SNPs

**Figure 3.**
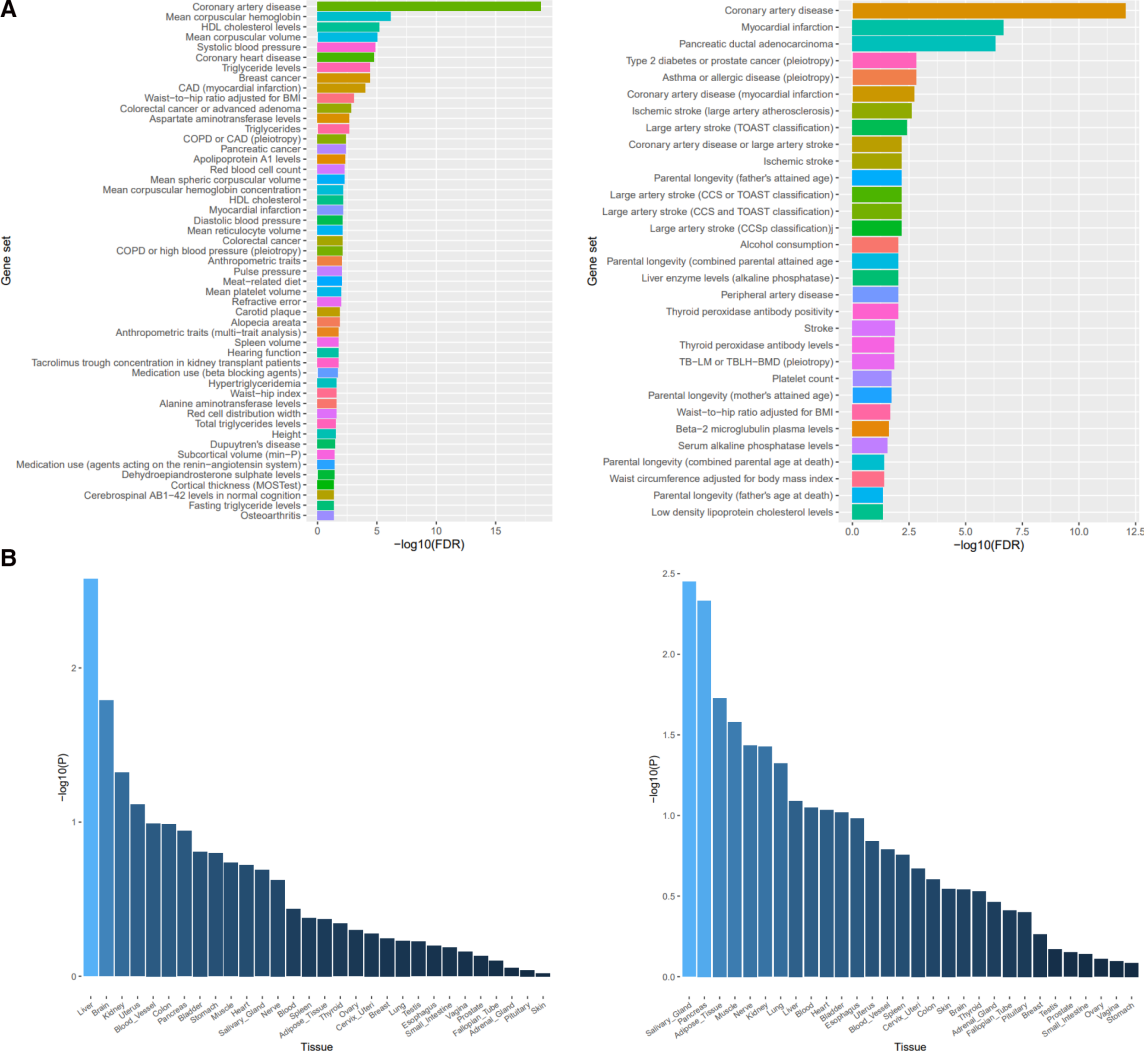
**Gene-set enrichment and tissue specificity analyses. A**, Comparison of gene-set enrichment of candidate joint susceptibility genes mapped to opposing-direction (**left**) and same-direction (**right**) lead SNPs (single-nucleotide polymorphisms). **B**, Comparison of tissue specificity of candidate joint susceptibility genes mapped opposing-direction (**left**) and same-direction (**right**) lead SNPs. AB1-42 indicates cerebrospinal fluid Aβ1–42 levels; BMI, body mass index; CAD, coronary artery disease; COPD, chronic obstructive pulmonary disease; CCS, causative classification of stroke; FDR, false discovery rate; HDL, high-density lipoprotein; MOSTest, multivariate omnibus statistic test; TB-LM, total-body lean mass; TBLH-BMD, total-body less head bone mineral density; and TOAST, Trial of Org 10172 in Acute Stroke Treatment.

Given that the majority (≈60%) of lead SNPs at colocalized pleiotropic loci had opposing effects across CAD and cancer risk, we then examined whether SNPs with opposing effects showed differential enrichment patterns as compared to those SNPs that acted in the same direction. We found that genes mapped to opposing-direction SNPs were more likely to be enriched for gene sets related to systolic blood pressure and blood cell traits (eg, mean corpuscular hemoglobin, mean corpuscular volume, mean platelet volume), whereas same-direction SNPs were more likely to be enriched for gene sets related to waist circumference adjusted for body mass index (Figure 3A). We found similar patterns of tissue expression across genes mapped to the same-direction and opposing-direction SNPs (Figure 3B).

### Multitrait Colocalization and Cis-Mendelian Randomization Identified Molecular Intermediates Associated With CAD and Cancer Risk

In multitrait colocalization analysis using HyPrColoc, there was evidence that 13 previously identified colocalized loci further colocalized with 30 measures of gene or protein expression. These included multitrait colocalization at 6 loci with a consistent direction of effect between CAD and cancer (*ABO*, *PGAP3*, *ANGPTL4*, *SREBF1*, *HAUS6*, and *SYNJ2BP*) and 7 with an opposing direction (*CDKN1A*, *RGS19*, *CALCRL*, *SF3A3*, *LAMC1*, *MYO9B*, and *MIA3*; Table S17). Overall, 11 of 13 multitrait colocalized loci were associated with tissue-specific or whole-blood gene expression, and 2 loci were associated with plasma protein expression. Candidate genes at multitrait colocalized loci have diverse functions, including roles in cell growth regulation (eg, *CDKN1A*), signal transduction (eg, *RGS19*), collagen transport (eg, *MIA3*), and lipid regulation or remodeling (eg, *ANGPTL4*, *SREBF1*, and *PGAP3*). In protein-protein interaction network analysis, 8 of 11 novel multitrait colocalized loci mapped to previously reported CAD or cancer susceptibility genes (Table S18). Findings from *cis*-Mendelian randomization analyses estimating the effect of genetically proxied molecular intermediates on CAD and site-specific cancer risk are presented in Table S19. Characteristics of genetic instruments for all molecular intermediates are presented in Table S20. F statistics for all genetic instruments were >10, suggesting that findings were unlikely to be influenced by weak instrument bias (Table S20).

### Phenome-Wide Association Study of Multitrait Colocalized Loci in UK Biobank

In PheWAS, we identified 1955 associations of lead SNPs at 13 multitrait colocalized loci with serum biomarkers, anthropometric traits, clinical measures, or plasma proteins measured in the UK Biobank. For example, rs2313171, the lead variant at the multitrait (CAD, colorectal cancer, whole blood, and tissue-specific *PGAP3* expression) colocalized *PGAP3* locus, was associated with 203 traits including fatty acid binding protein 9 (FDR *P*=4.91×10^−110^), white blood cell count (FDR *P*=9.41×10^−51^), aspartate aminotransferase (FDR *P*=1.23×10^−40^), and and HDL cholesterol levels (FDR *P*=1.66×10^−38^). Across all PheWAS, traits strongly associated (FDR *P*<0.05) with the largest number of loci were: apoA (8), HDL cholesterol (7), total cholesterol (6), and creatinine (6). Complete findings from PheWAS analyses are presented in Tables S21 through S33.

### Druggability and Drug Repurposing Evaluation

When referencing the druggability and drug development profiles of genes mapped to multitrait colocalized pleiotropic loci, 5 of 13 candidate joint susceptibility genes were approved, investigational, or literature-reported targets of medications or were considered druggable (Table S34). The majority of these drugs do not currently have CAD or cancer-related indications. Genes mapping to approved or investigational drugs included *CALCRL* (target of several medications used to manage migraine disorder), *LAMC1* (target of ocriplasmin, used to treat vitreomacular traction), and *ANGPTL4* (investigational target for hypertriglyceridemia).

## Discussion

Our comprehensive genome-wide pleiotropy and colocalization analysis of coronary artery disease and 4 common cancers identifies 60 susceptibility loci associated with both conditions, most of which are newly reported as pleiotropic loci. Among these, 35 loci showed opposing effects on CAD and cancer risk, providing insights into mechanisms that may protect against one condition while increasing susceptibility to the other. Gene-set enrichment and tissue-specificity analysis highlighted distinct biological processes underlying pleiotropic loci with shared versus opposing effects, including distinct effects on blood pressure, blood cell traits, and waist circumference. We also found evidence that the effects of 13 colocalized pleiotropic loci were mediated via differential expression of neighboring candidate genes, enhancing mechanistic understanding of pathways contributing to joint disease liability. Phenome-wide association analysis in the UK Biobank further revealed these 13 loci were enriched for associations with large numbers of clinical traits and serum biomarkers, most notably apoA, HDL cholesterol, and creatinine. Five of these colocalized pleiotropic loci were mapped to genes encoding approved, investigational, or druggable targets, including *CALCRL*, *LAMC1*, and *ANGPTL4*, suggesting opportunities for targeted interventions. These findings clarify shared genetic and molecular mechanisms contributing to CAD and cancer comorbidities, highlight potential discordant effects of genetic and molecular pathways on each condition, and can inform drug target prioritization for dual disease prevention along with pharmacovigilance strategies for existing medications for these leading global causes of mortality.

Our findings highlight several known joint susceptibility loci for CAD and cancer, including *FKBP5*, *CUX2*, and *LMOD1*, validating the pleiotropy analysis approach employed by PLACO. We also identified >40 novel pleiotropic loci for CAD and cancer, the majority of which had opposing effects across conditions. For example, we found evidence that arterial *CALCRL* expression, CAD, and breast cancer susceptibility colocalized at the *CALCRL* locus. *CALCRL* encodes calcitonin receptor–like, a neuropeptide receptor involved in blood pressure regulation, cell proliferation, and apoptosis.^45^ In Mendelian randomization analysis, arterial *CALCRL* expression was associated with an increased risk of breast cancer but a reduced risk of CAD. Consistent with these opposing directions of effect, in vivo calcitonin receptor-like inhibition has been shown to reduce tumor growth in solid tumors but to increase atherosclerotic lesions.^46,47^ Calcitonin receptor-like antagonists have recently emerged as effective medications for the treatment of migraine disorder.^48–50^ Our findings suggesting potential opposing roles of calcitonin receptor-like in CAD and breast cancer development support continued safety monitoring of calcitonin receptor-like antagonists to evaluate the potential consequences of their long-term use.

We also found evidence of colocalization of whole-blood *CDKN1A* expression, CAD, and breast cancer susceptibility at the *CDKN1A* locus and that whole-blood *CDKN1A* expression increased the risk of CAD but lowered the risk of breast cancer. *CDKN1A* encodes p21, a potent cyclin-dependent kinase inhibitor that has been reported to have dual effects in breast cancer as both a key mediator of p53-dependent cell cycle arrest and an inhibitor of cell apoptosis.^51,52^ p21 has also been shown to mediate postnatal cardiomyocyte cell cycle arrest, facilitate the development of cardiac hypertrophy, and regulate LPS-induced cardiac dysfunction.^53–55^ Our findings suggest a potential dual role of CDKN1A across breast cancer and CAD, warrant further functional validation to elucidate potential mechanisms contributing to both conditions.

In addition, we found evidence that shared CAD and colorectal cancer signals in the *ANGPTL4* gene with the same direction of effect were mediated via circulating ANGPTL4 concentrations. ANGPTL4, a key regulator of triglyceride clearance via lipoprotein lipase inhibition, is an emerging therapeutic target for hypertriglyceridemia and has been implicated in colorectal tumourigenesis in in vitro and in vivo studies.^56–59^ Though further functional and experimental validation is required, our genetic findings tentatively support the pharmacological inhibition of ANGPTL4 as a potential strategy for the dual prevention of CAD and colorectal cancer.

That lead SNPs from 35 of 60 colocalized pleiotropic loci conferred effects in opposing directions across CAD and cancer risk raises the possibility that biological pathways contributing to one disease may protect against the other. Along with differences in tissue expression patterns, we found that colocalized pleiotropic loci with effects on CAD and cancer risk in opposing directions were more strongly enriched for blood pressure-related traits, blood cell traits, whereas same-direction loci were more strongly enriched for waist circumference. These findings align with conventional and genetic epidemiological evidence suggesting the potential adverse effects of select antihypertensive agents (ie, ACE [angiotensin-converting enzyme inhibitors]) on cancer risk, though mechanisms accounting for these associations remain unclear.^60,61^ Further functional work is required to validate these findings to elucidate mechanisms accounting for opposing effects across CAD and cancer risk.

Strengths of this analysis include the comprehensive assessment of shared genetic and molecular signals across liability to CAD and cancer risk in humans. We leveraged large-scale genetic association data from various GWAS consortia of CAD and site-specific cancer risk, enhancing statistical power and precision of estimates of joint disease liability. By integrating large-scale transcriptomic data from Genotype-Tissue Expression and eQTLgen and proteomic data from deCODE analyses into multitrait colocalization, we identified putative molecular mechanisms mediating the effects of shared genetic loci on CAD and cancer risk at 12 loci. Finally, the integration of phenome-wide association study analysis and gene set enrichment provided further insight into potential mechanisms contributing to CAD and cancer comorbidities.

There are several limitations to this analysis. First, relevant transcriptomic and proteomic data were not available for genes mapped to the majority of colocalized pleiotropic loci, restricting our ability to explore the potential mediating effect of these molecular intermediates. Second, the lack of sex-stratified CAD GWAS data available limited our ability to further explore shared susceptibility loci with breast and prostate cancer. However, to date, analyses exploring sex-specific genetic associations with CAD susceptibility have found limited evidence of differential genetic effects by sex.^21,62^ Third, our analyses were primarily performed in individuals of European ancestry and, therefore, the generalizability of these findings to non-European populations is unclear. Fourth, our findings suggesting shared genetic signals across CAD and cancer risk could reflect 1 or more of the following interpretations: (1) biological pleiotropy (ie, both CAD and cancer risk are independently influenced by the same risk factor that is on distinct causal pathways), (2) mediated pleiotropy (ie, both CAD and cancer risk are influenced by the same risk factor that is on the same causal pathway), and (3) spurious pleiotropy (ie, a risk factor only influences 1 trait, but appears to influence the other trait via, for example, misclassification or ascertainment bias or via genetic confounding due to high LD).^63^ In our analysis, spurious pleiotropy via genetic confounding should be minimized with the use of colocalization as a sensitivity analysis. However, we cannot rule out the possibility that mediated pleiotropy reflects a primary effect of a risk factor on the development of CAD, which then subsequently influences cancer risk via an effect of a medication used to treat CAD on cancer risk, rather than an effect of CAD itself. This is supported by evidence from both genetic and conventional observational studies that various cardiometabolic medications may influence cancer risk.^60,64–66^ Fifth, as advised by the authors of PLACO, we removed variants with Z^2^>80 to minimize bias from extremely large effect sizes. Although this analytical step was implemented to reduce potential bias, it is possible that the exclusion of variants with Z^2^>80 could have removed variants that have large effects on both CAD and cancer risk. Sixth, our restriction of pleiotropy exploration to protein-coding genes ignored the potential role of functional RNA molecules (eg, noncoding RNA, long noncoding RNA) and regulatory elements in disease causation. Finally, although our findings provide human genetic support for putative joint susceptibility loci for CAD and cancer risk, experimental perturbation in in vivo models and clinical trials in humans are required to establish causality of candidate joint susceptibility genes in CAD and cancer risk.

## Conclusions

Our comprehensive genome-wide pleiotropy analysis across CAD and cancer risk identified 60 loci jointly influencing both conditions, most of which are novel and show opposing effects on CAD and cancer. Through gene set enrichment and tissue-specificity analysis, we identify distinct biological pathways enriched among pleiotropic loci with effects in the same direction as compared with opposing directions, and through integration of proteomic and transcriptomic data and phenome-wide association study analysis, we highlight putative molecular mechanisms linking joint susceptibility loci to CAD and cancer. Finally, the identification of approved and investigational drug targets associated with both CAD and cancer offers valuable insights for developing pharmacological approaches for dual disease prevention and for anticipating potential adverse and divergent effects of these medications on CAD and cancer risk.

## Article Information

### Sources of Funding

J. Yarmolinsky and I. Tzoulaki are supported by the National Institute for Health and Care Research Imperial Biomedical Research Center. D. Wang is supported by the UK Academy of Medical Sciences (APR7_1002), UK Medical Research Council (MR/Z505468/1), and UK Engineering and Physical Sciences Research Council (EP/V029045/1).

### Disclosures

None.

### Supplemental Material

Tables S1–S34

Figures S1–S3

Major Resources Table

STREGA Reporting Guidelines Checklist
